# Domain-specific mutations in *unc-6/Netrin* differentially affect dorsal-ventral axon pathfinding in *Caenorhabditis elegans*

**DOI:** 10.1242/bio.062822

**Published:** 2026-09-16

**Authors:** Kelsey M. Hooper, Scott G. Clark, Erik A. Lundquist

**Affiliations:** ^1^ Department of Molecular Biosciences, Program in Molecular, Cellular, and Developmental Biology, KU Center for Genomics, University of Kansas, Lawrence, KS 66045, USA; ^2^ Molecular Neurobiology Program, Department of Pharmacology, Skirball Institute, NYU School of Medicine, New York, NY 10016, USA. (Retired)

**Keywords:** *Caenorhabditis elegans*, UNC-6/Netrin, Axon pathfinding

## Abstract

UNC-6/Netrin is a conserved regulator of dorsal-ventral axon and cell migrations. Here, we identified missense mutations in distinct UNC-6 domains and assessed their roles in dorsal VD/DD motor axon guidance and ventral anterior ventral microtubule (AVM) axon guidance. A missense mutation in a conserved residue of the laminin N-terminal (LN) domain (G289D) resulted in dorsal and ventral axon guidance defects similar to the *unc-6* null. A distinct missense mutation in the LN domain (S120F) strongly perturbed ventral AVM axon guidance with minimal effects on dorsal VD/DD axon guidance. Mutations altering cysteine residues involved in disulfide bonding in the epidermal growth factor (EGF) domains were analyzed. EGF1(C321G) and EGF2(C347Y) caused both ventral and dorsal axon guidance defects, whereas EGF3(C410Y) specifically disrupted dorsal axon guidance. The crystal structure of UNC-6 shows conserved N-linked glycosylation at N114 and N128. These sites were not solely required for axon guidance, but mutations interacted genetically with *unc-40* and *unc-5* mutations, indicating that these residues have a role in UNC-6 signaling. Our results reveal the effects of UNC-6 domains on dorsal-ventral axon guidance and will inform studies on how these distinct UNC-6 domains interact with guidance receptors (e.g. UNC-40/DCC and UNC-5) and other extracellular molecules to mediate dorsal-ventral axon guidance.

## Teaser

**Summary:** New mutations in UNC-6/Netrin reveal roles of distinct domains in dorsal-ventral axon pathfinding. These studies contribute to understanding how UNC-6/Netrin might interact with distinct guidance receptors to mediate specific guidance events.

## INTRODUCTION

UNC-6/Netrin and its receptors UNC-40/DCC and UNC-5 control dorsal-ventral axon guidance in *Caenorhabditis elegans* (Hedgecock et al., 1990; Ishii et al., 1992; Serafini et al., 1994; Norris and Lundquist, 2011; Boyer and Gupton, 2018). Classically, the UNC-40/DCC receptor was thought to mediate ventral growth via attraction to UNC-6 (Chan et al., 1996; Keino-Masu et al., 1996; Kolodziej et al., 1996; Deiner et al., 1997), and the UNC-5 receptor was thought to control dorsal growth by repulsion from UNC-6 (Leung-Hagesteijn et al., 1992; Hamelin et al., 1993). Analysis of growth cone outgrowth in Netrin signaling mutants in *C. elegans* has led to a revision of this view and indicates that UNC-40/DCC and UNC-5 are both involved in ventral and dorsal axon guidance. In the statistically oriented asymmetric localization model of growth toward Netrin, the protrusive activity of UNC-40/DCC is localized ventrally by UNC-6 in the hermaphrodite-specific neuron (HSN), and UNC-5 focuses and maintains this polarity so that the HSN axon extends ventrally toward the UNC-6 source (Kulkarni et al., 2013; Yang et al., 2014; Limerick et al., 2017). In the polarity/protrusion model of dorsal growth away from UNC-6, Netrin first polarizes the VD growth cone through UNC-5 so that the pro-protrusive activity of UNC-40/DCC is localized dorsally, and then UNC-6 and UNC-5 maintain this polarity as the growth cone protrudes and grows dorsally away from the UNC-6/Netrin source (Norris and Lundquist, 2011; Norris et al., 2014; Gujar et al., 2018; Mahadik and Lundquist, 2023; Hooper et al., 2025).

UNC-6/Netrin is similar to the N-terminus of laminins and consists of a LN domain, three laminin-like epidermal growth factor (EGF) repeats, and a Netrin-specific C-terminal (NCD) domain (Ishii et al., 1992). Previous structure-function studies using domain deletions and mutations indicate that the LN domain and the EGF1 and EGF2 domains are required for both dorsal and ventral axon guidance and that the EGF3 domain is required predominantly for dorsal axon guidance (Hedgecock et al., 1990; Wadsworth and Hedgecock, 1996; Lim and Wadsworth, 2002; Norris and Lundquist, 2011). The NCD is not involved in dorsal or ventral axon guidance activity but rather acts to suppress ectopic branching (Lim et al., 1999; Lim and Wadsworth, 2002; Wang and Wadsworth, 2002). The UNC-5 extracellular immunoglobulin-like domain 2 (IG2) is predicted to physically interact with the UNC-6 LN and EGF1 domains, consistent with their roles in dorsal growth (Priest et al., 2024). Heparin binding at the IG1 and IG2 domains of UNC-5 stabilizes the UNC-5-UNC-6 complex and excludes interaction with UNC-40 (Priest et al., 2024). Mammalian DCC interacts with Netrin1 at the LN, EGF2, and EGF3 domains (Finci et al., 2014; Xu et al., 2014), consistent with functional studies of *C. elegans* UNC-6 and UNC-40 (Priest et al., 2024).

Here, we report the identification, analysis, and sequencing of 14 new *unc-6* alleles. Of these, four are missense mutations in specific UNC-6 domains. We characterized how these missense mutations affected the dorsal axon guidance of the ventral and dorsal D-type motor neurons (VD/DD) and the ventral axon guidance of the anterior ventral microtubule (AVM) neuron. A glycine 289-to-aspartic acid (G289D) substitution in the LN domain strongly disrupted both dorsal and ventral axon guidance, consistent with previous results (Lim and Wadsworth, 2002). A serine 120-to-phenylalanine (S120F) substitution in the LN domain was hypomorphic and predominantly perturbed ventral AVM axon guidance with minimal disruption of dorsal VD/DD axon guidance. Mutations of cysteines that make disulfide bonds in the EGF1(C321G) and EGF2(C347Y) disrupted both dorsal and ventral axon guidance, although to a lesser extent than the null *unc-6(ev400)*. As reported previously (Norris and Lundquist, 2011), the *unc-6(e78)* mutation that alters a disulfide-forming cysteine in the EGF3 domain (C410Y) strongly disrupted dorsal VD/DD axon guidance yet only partially disrupted ventral AVM axon guidance. In sum, our studies identified missense mutations that specifically affect the LN, EGF1, and EGF2 domains and a novel S120F mutation in the LN domain that predominantly altered ventral AVM axon guidance. Our investigation using endogenous mutations complements previous structure-function studies, with domain deletion rescuing transgenes (Lim and Wadsworth, 2002).

The crystal structure of UNC-6 from protein produced in insect cells shows that UNC-6 is glycosylated at N114 and N128 (Priest et al., 2024). To test the role of these residues of UNC-6 in dorsal-ventral (DV) axon guidance, we changed N114 and N128 to alanine using Cas9 genome editing. We did not detect defects in VD/DD or AVM axon guidance in single and double mutants. However, we found via genetic interactions with *unc-40* and *unc-5* that the UNC-6 function was modestly reduced when these asparagine residues were altered.

## RESULTS

### Isolation and analysis of *unc-6* alleles

We recovered 14 new *unc-6* alleles in genetic screens for axon guidance mutants (see Materials and Methods). Each failed to complement the null *unc-6(ev400)* allele for the uncoordinated (Unc) phenotype and was rescued for Unc by the *unc-6(+)* transgene *lqEx1241* (see Materials and Methods). We identified sequence changes in the *unc-6* gene in the mutants using Nanopore amplicon sequencing (see Materials and Methods). *zd17*, *zd116*, *zd146*, and *zd154* contain missense mutations (Table 1 and Fig. 1). The mutation in *zd17* changed serine 120 to phenylalanine, and the mutation in *zd146* changed glycine 289 to aspartic acid, both in the LN domain. Both sites are conserved in Netrin and laminin sequences across phylogeny including vertebrates (Shaw et al., 2021). Indeed, the equivalent of UNC-6 glycine 289 is affected by pathogenic missense mutations in human laminin α2 (limb-girdle-type dystrophy) (Gavassini et al., 2011). Mutation of *Drosophila* laminin β1 at this position led to cardiac development defects (Hollfelder et al., 2014).

**Fig. 1.**
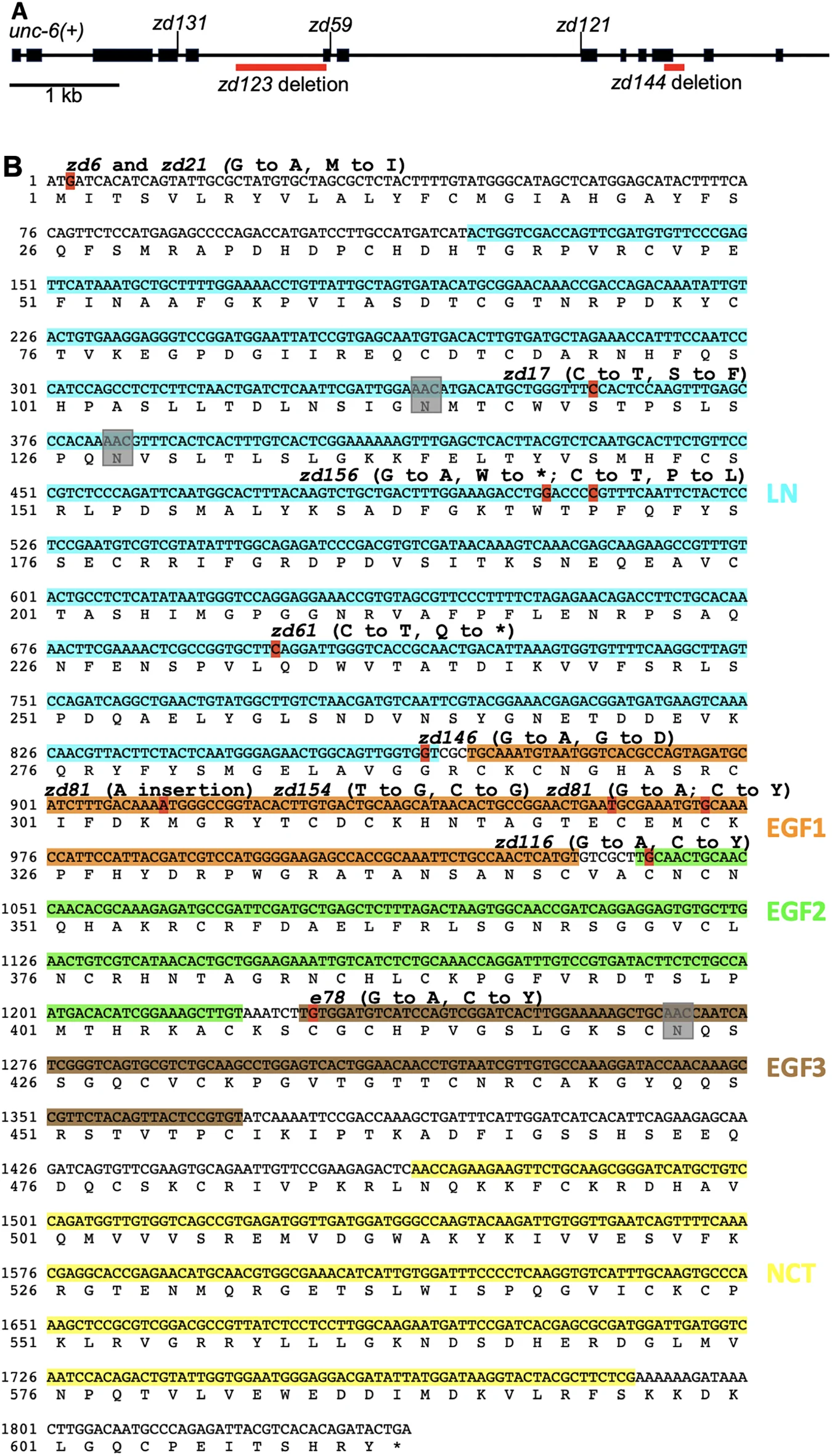
**The *unc-6* locus and new *unc-6* alleles.** (A) A diagram of the *unc-6* locus. Exons are represented by boxes, and introns by lines. The *zd123* and *zd144* deletions are shown, and the *unc-6* mutations affecting splice sites are indicated. (B) The *unc-6* cDNA sequence. The LN domain is highlighted in blue; the first EGF domain (EGF1) in orange; EGF2 in green; EGF3 in brown; and the Netrin C-terminal domain in yellow. The locations of new *unc-6* alleles are shown above the sequence and indicated in red. The three conserved potential N-glycosylation sites are highlighted by gray boxes.

**Table 1.** 14 new unc-6 alleles

Allele	Sequence^1^	Change
LN domain mutations		
*zd17*	TGCTGGGTTTCCACTCCAAGT TGCTGGGTTTTCACTCCAAGT	S120F
*zd146*	AGTTGGTGGTCGCTGCAAATG AGTTGGTGATCGCTGCAAATG	G289D
EGF domain mutations		
*zd154*	GGAACTGAATGCGAAATGTGC GGAACTGAAGGCGAAATGTGC	EGF1 C321G
*zd116*	tccagCTTGCAACTGCAACCA tccagCTTACAACTGCAACCA	EGF2 C347Y
*e78*	acttaagCTTGTGGATGTCAT acttaagCTTATGGATGTCAT	EGF3 C410Y
Initiator methionine mutations		
*zd6*	cacacaaacATGATCACATCA cacacaaacATAATCACATCA	M1I
*zd21*	cacacaaacATGATCACATCA cacacaaacATAATCACATCA	M1I
Nonsense mutations		
*zd61*	CCGGTGCTTCAGGATTGGGTC CCGGTGCTTTAGGATTGGGTC	Q234*
*zd156*	AGACCTGGACCCCGTTTCAAT AGACCTGAACCCTGTTTCAAT	W168* P170L
Frameshift mutation		
*zd81*	CTTTGACAAAATGGGCCGGgt CTTTGACAAAAATGGGCCGGgt TGCGAAATGTGCAAACCATTC TGCGAAATGTACAAACCATTC gacggggggggggggggggag gacggggggggggggggggggggag	An insertion in codon 305, frameshift C324Y Intron insertion
Deletion mutations		
*zd123*	ttcactttt---ct---CTCTTTAGA^2^	924 bp deletion, CT insertion
*zd144*	CACCGAGAAC---ttttgtttga^3^ gacggggggggggggggggag gacggggggggggggggggggggag	132 bp deletion Intron insertion
Splice site mutations		
*zd59*	AGTGGCAACCGgttagccatc AGTGGCAACCGattagccatc	5′ splice
*zd131*	ATGGGCCGGgtaagcactcac ATGGGCCGGataagcactcac	5′ splice
*zd121*	ccacttaagCTTGTGGATGTC ccacttaaaCTTGTGGATGTC	3′ splice

^1^*Wild-type* sequence is shown above, and mutant sequence is shown below, with the change in red and underlined.

^2^*zd123* breakpoints are before and after the hyphens, with a CT insert.

^3^*zd144* breakpoints are before and after the hyphens.

*zd154* changed cysteine 321 to glycine in the first EGF domain, and *zd116* changed cysteine 347 to tyrosine in the second EGF domain. The *unc-6(e78)* allele was previously shown to change cysteine 410 to tyrosine in the third EGF domain. The cysteines affected by *zd154* (EGF1), *zd116* (EGF2), and *e78* (EGF3) are each involved in disulfide bonds that contribute to the structure of the EGF domains (Fig. 2A-C).

**Fig. 2.**
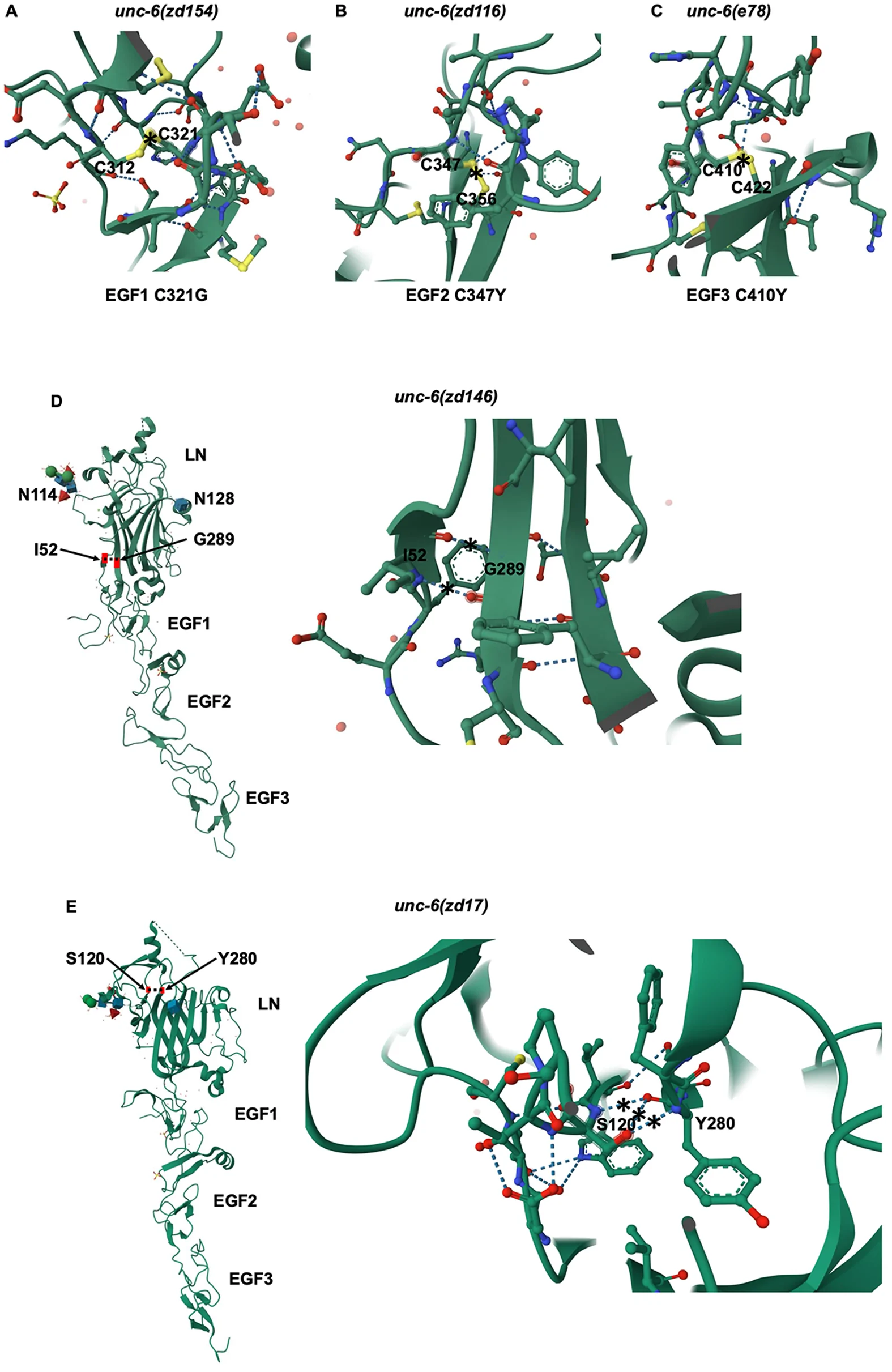
**Crystal structures of UNC-6 regions affected by new *unc-6* missense mutations.** Images were generated by Mol* from the X-ray crystal structure of the UNC-6 LN and EGF domains in the Protein Data Bank (Berman et al., 2000; Priest et al., 2024) (pdb00008EDK). (A) EGF1 residue cysteine 321, changed to a glycine in *unc-6(zd154)*, normally makes a disulfide bond with cysteine 312 (asterisk). (B) EGF2 residue cysteine 347, changed to a tyrosine in *unc-6(zd116)*, normally makes a disulfide bond with cysteine 356 (asterisk). (C) EGF3 residue cysteine 410, changed to a tyrosine in *unc-6(e78)*, normally makes a disulfide bond with cysteine 422 (asterisk). (D) In the LN domain, glycine 289, changed to aspartic acid in *unc-6(zd146)*, makes two hydrogen bonds with isoleucine 52 in an adjacent β-strand (dotted line and asterisks). The potentially N-glycosylated residues, asparagine 114 and asparagine 128 in the LN domain, are also indicated. (E) Serine 120 in the LN domain, changed to phenylalanine in *unc-6(zd17)*, makes three hydrogen bonds with tyrosine 380 in an adjacent β-strand in the ‘jellyroll’ β-barrel structure.

The remaining ten *unc-6* alleles harbor mutations that are predicted to produce nonfunctional UNC-6 protein because of failure to initiate translation, premature termination of translation, or aberrant mRNA splicing. *zd6* and *zd21* contain the same missense mutation that converts the initiator methionine to isoleucine (1MI) (Table 1 and Fig. 1). There is an in-frame ATG at position 16, which, if used, would result in a molecule lacking a signal peptide. *zd61* and *zd156* contain nonsense mutations that introduce premature stop codons (Table 1 and Fig. 1). *zd156* had a second C-to-T mutation resulting in a P170L missense mutation five bases downstream of the nonsense mutation (Table 1). *zd81* contains an A residue insertion in codon 305, resulting in a frameshift and an in-frame premature stop codon six codons downstream. *zd81* also contains a missense mutation (C324Y) in the *wild-type* reading frame downstream of the frameshift and an insertion of four G residues in intron 7. *zd123* contains a 924 bp deletion with a 2 bp insertion that removes 47 bp of coding sequence in addition to intron sequence (Table 1 and Fig. 1). *zd144* contains a 132 bp deletion that removes 94 bases of the coding region as well as intron sequence (Table 1 and Fig. 1). *zd144* also has the insertion of four G residues in intron 7, identical to *zd81. zd59*, *zd121*, and *zd131* contain point mutations predicted to disrupt splicing of *unc-6* mRNA: *zd59* and *zd131* altered an invariant G in the 5′ splice donor sequence, and *zd121* changed an invariant G in the 3′ splice acceptor sequence (Fig. 1 and Table 1).

### Dorsal-ventral axon guidance defects in LN domain *unc-6* mutants

The axons of the VD and DD motor neurons are a model for dorsal growth cone migration away from the UNC-6 source in the ventral nerve cord (VNC). The 19 VD and DD motor neurons extend axons dorsally from the VNC to the dorsal nerve cord (DNC). In wild type, we observed an average of 16.21 commissures due to fasciculation of some axons into single commissures (Table 2 and Fig. 3A). Null *unc-6(ev400)* mutants displayed severe defects in dorsal VD/DD axon guidance, with very few axons reaching the DNC (an average of 0.02 per animal, or only two observed in 100 animals) and an average of only 6.22 per animal showing any extension dorsally from the VNC (Table 2 and Fig. 3B).

**Fig. 3.**
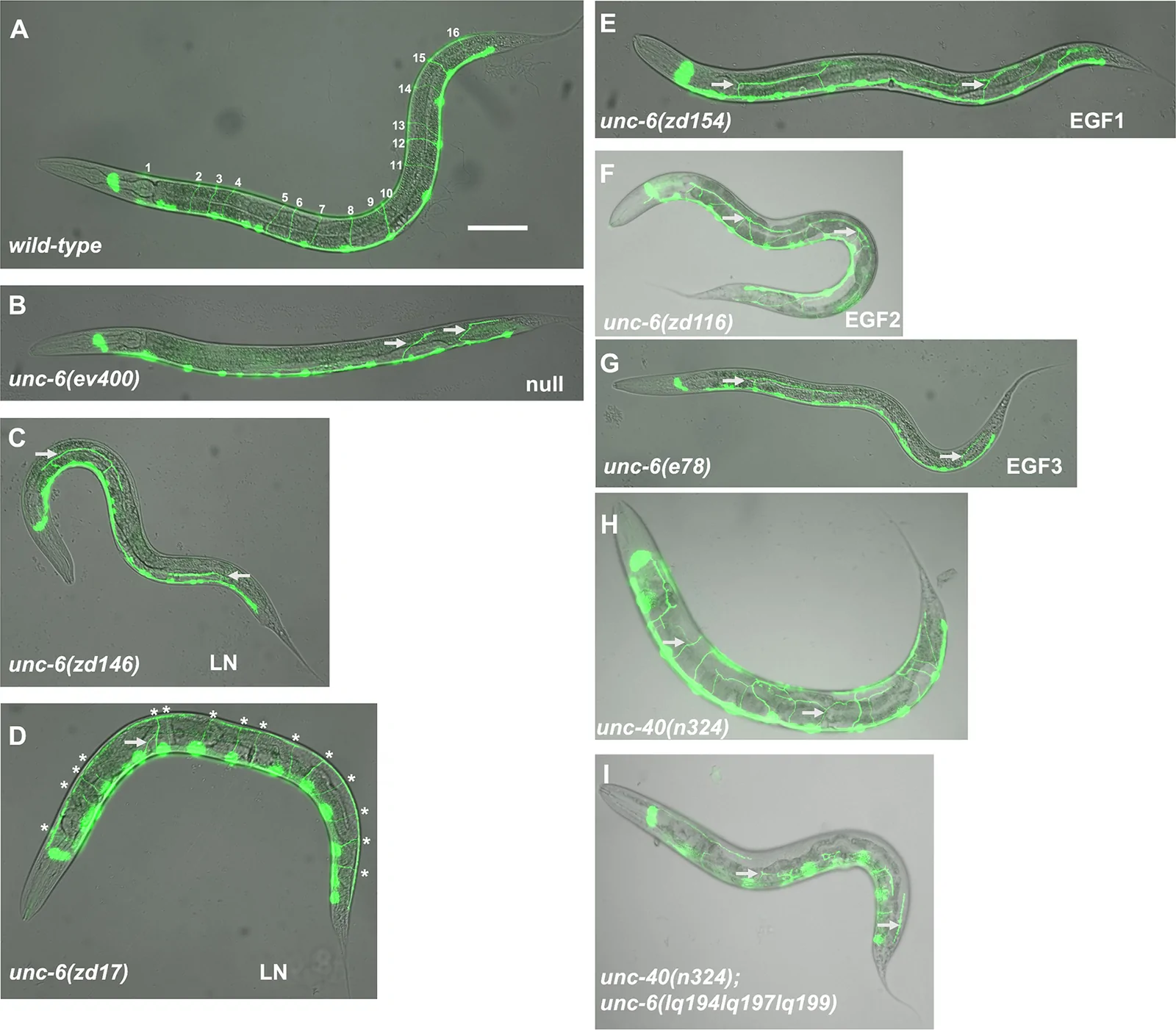
**Dorsal VD/DD axon guidance defects in *unc-6* mutants.** Micrographs of the *juIs76[Punc-25::gfp]* transgene expressed in the GABAergic motor neurons with a merged differential interference contrast micrograph are shown. The anterior is to the left and dorsal is upward. The scale bar in A indicates 50 μm for all micrographs. The domain affected by each mutant is indicated. (A) A wild-type animal displaying 16 visible VD/DD commissural tracts from the VNC to the DC (numbers). (B) An *unc-6(ev400)* null mutant. Arrows point to axons that emerged from the VNC, but none reached the DNC. (C) An *unc-6(zd146)* mutant with axons emerging from the VNC (arrows) but none reaching the DNC. (D) An *unc-6(zd17)* mutant showed 15 normal VD/DD axon commissures (asterisks) with one misguided axon (arrow). (E) An *unc-6(zd154)* mutant with some misguided axons emanating from the VNC (arrows). (F) An *unc-6(zd116)* mutant with many axons emanating from the VNC but not reaching the DNC (arrows). (G) An *unc-6(e78)* mutant with some axons emanating from the VNC (arrows). (H) An *unc-40(n324)* mutant with defective VD/DD axon guidance (arrows), many of which extend away from the VNC and reach the DNC. (I) An *unc-40(n324); unc-6(lq194lq197lq199)* mutant with severe VD/DD axon guidance defects (arrows), with few axons extending away from the VNC.

**Table 2.** VD/DD and AVM axon guidance defects

Genotype	Average # VD/DD emerging	Average # VD/DD reaching	% defective ventral AVM axon guidance
*Wild-type*	16.02	15.95	0
*unc-6(ev400)* Null	6.22	0.02	72
*unc-6(zd146)* LN	5.57	0.04	67
*unc-6(zd17)* LN	15.82	9.94	30
*unc-6(zd154)* EGF1	6.05	0.02	35
*unc-6(zd116)* EGF2	12.80	1.78	18
*unc-6(e78)* EGF3	4.54	0.03	8
*unc-6(zd144)* C-terminal deletion	8.41	0.04	45
*unc-6(lq194) N114A*	15.98	15.92	0
*unc-6(lq196) N128A*	16.00	15.91	0
*unc-6(lq200) N423A*	15.87	15.78	0
*unc-6(lq194lq197) N114A, N128A*	16.03	15.91	0
*unc-6(lq194lq197lq199) N114A, N128A, N423A*	15.76	15.58	0
*unc-40(n324)*	15.76	9.88	11
*unc-40(n324); unc-6(N114A)*	15.64	7.13	ND
*unc-40(n324); unc-6(N128A)*	15.61	7.23	ND
*unc-40(n324); unc-6(N423A)*	15.37	4.66	ND
*unc-40(n324); unc-6(N114A N128A)*	15.70	6.41	ND
*unc-40(n324); unc-6(N114A N128A N423A)*	15.13	3.60	17
*unc-5(ev480)*	10.72	6.61	0
*unc-5(ev480); unc-6(N114A)*	13.34	4.06	2
*unc-5(ev480); unc-6(N128A)*	14.04	4.68	1
*unc-5(ev480); unc-6(N423A)*	13.57	4.82	1
*unc-5(ev480); unc-6(N114A N128A)*	13.48	4.54	3
*unc-5(ev480); unc-6(N114A N128A N423A)*	9.76	2.82	5
*unc-5(e791)*	0.25	0	4
*unc-5(e791); unc-6(N114A N128A N423A)*	ND	ND	7
Statistical comparisons	ANOVA	ANOVA	Fisher's exact test
*zd146 versus ev400*	NS	NS	NS
*zd17 versus ev400*	*P<*0.0001	*P<*0.0001	*P<*0.0001
*zd154 versus ev400*	NS	NS	*P<*0.0001
*zd116 versus ev400*	*P<*0.0001	*P<*0.0001	*P<*0.0001
*zd144 versus ev400*	*P<*0.0001	NS	*P*=0.0002
*zd116 versus zd154*	*P<*0.0001	*P<*0.0001	*P*=0.0100
*zd116 versus e78*	*P<*0.0001	*P<*0.0001	*P*=0.0568
*e78 versus ev400*	*P<*0.0001	NS	*P<*0.0001
*e78 versus zd17*	*P<*0.0001	*P<*0.0001	*P<*0.0001
*lq194 versus wild type*	NS	NS	NS
*lq196 versus wild type*	NS	NS	NS
*lq200 versus wild type*	NS	NS	NS
*lq194lq197 versus wild type*	NS	NS	NS
*lq194lq197lq199 versus wild type*	NS	*P*=0.0261	NS
*n324; lq194 versus n324*	NS	*P<*0.0001	ND
*n324; lq196 versus n324*	NS	*P<*0.0001	ND
*n324; lq200 versus n324*	NS	*P<*0.0001	ND
*n324; lq194lq197 versus n324*	NS	*P<*0.0001	ND
*n324; lq194lq197lq199 versus n324*	NS	*P<*0.0001	NS
*ev480; lq194 versus ev480*	*P<*0.0001	*P<*0.0001	NS
*ev480; lq196 versus ev480*	*P<*0.0001	*P<*0.0001	NS
*ev480; lq200 versus ev480*	*P<*0.0001	*P<*0.0001	NS
*ev480; lq194lq197 versus ev480*	*P<*0.0001	*P<*0.0001	NS
*ev480; lq194lq197lq199 versus ev480*	*P*=0.0037	*P<*0.0001	*P*=0.0594
*e791 versus e791; lq194lq197lq199*	ND	ND	NS

The axon of the AVM mechanosensory neuron is a model for ventral axon growth toward the UNC-6 source in the VNC. The AVM cell body resides laterally and extends an axon ventrally to the VNC (Fig. 4A). In *unc-6(ev400)* null mutants, the AVM axon failed to extend ventrally in 72% of animals and instead extended laterally toward the anterior (Table 2 and Fig. 4B).

**Fig. 4.**
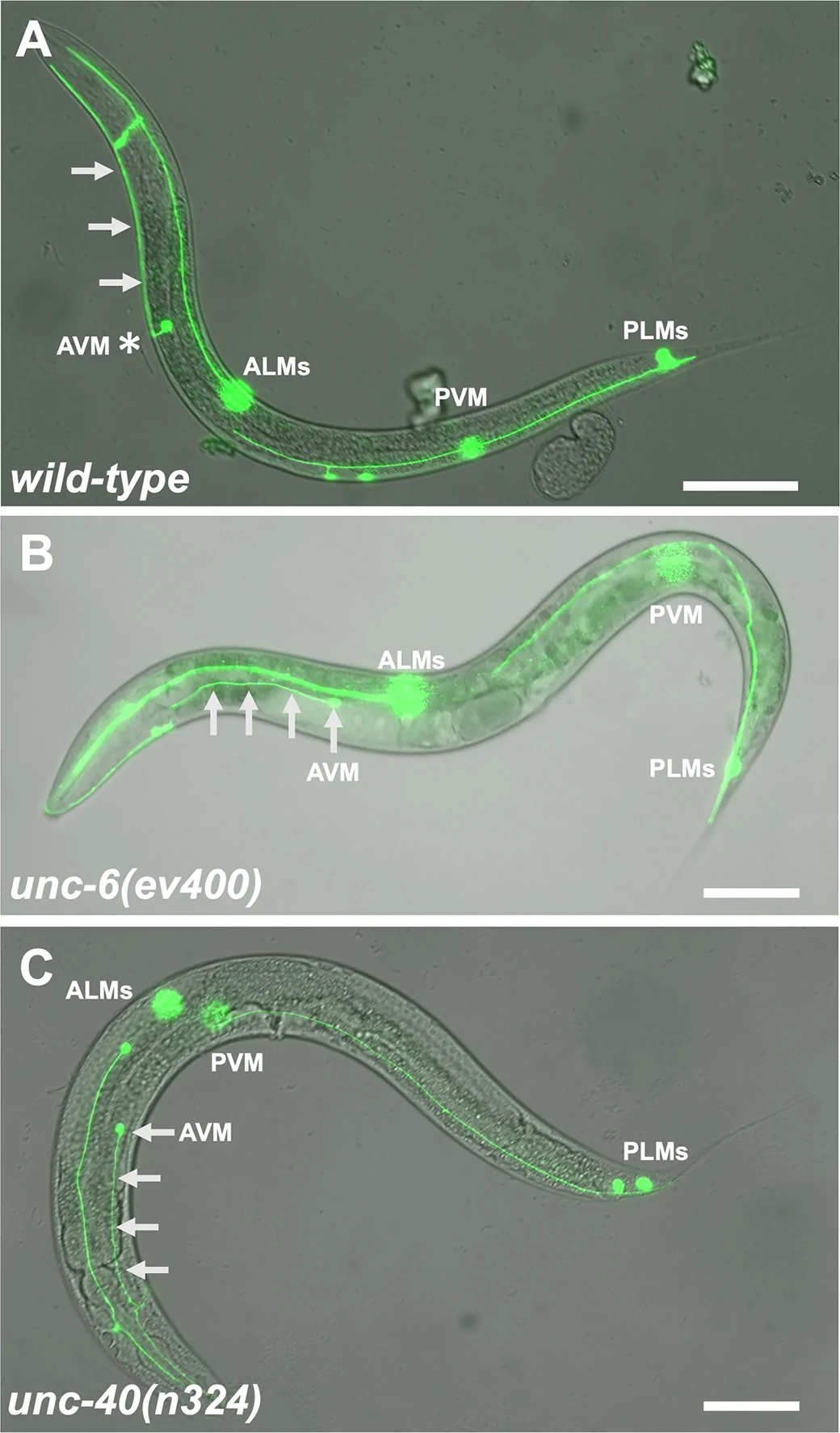
**Ventral AVM axon guidance defects.** Merged GFP fluorescence and DIC micrographs are shown in Fig. 3. Scale bars: 50 μm. A and B are the *zdIs5[Pmec-4::gfp]* transgene, and C is the *zdIs4[Pmec-4::gfp] zdIs4[Pmec-4::gfp]* transgene, both expressed in the mechanosensory neurons. The positions of the AVM cell bodies are indicated, along with those of the anterior lateral microtubule neurons (ALMs), posterior lateral microtubule neurons (PLMs), and the posterior ventral microtubule neuron (PVM), which are occasionally out of focus. The anterior is to the left and dorsal is upward. (A) A *wild-type* animal in which the AVM axon extends directly ventrally from the cell body (asterisk). The AVM axon then runs anteriorly in the VNC (arrows). (B) An *unc-6(ev400)* animal in which the AVM axon fails to extend ventrally and instead extends anteriorly out of the AVM cell body along the lateral body wall (arrows). (C) An *unc-40(n324)* animal in which an AVM axon fails in ventral extension and instead extends anteriorly along the lateral body wall (arrows). The PVM cell body is also anteriorly displaced due to the previously described defective migration of the QL neuroblast and descendants in *unc-40* mutants.

We scored axon defects in *unc-6(zd17)* and *unc-6(zd146)* missense mutations in the LN domain. *unc-6(zd146)* animals were strongly uncoordinated and displayed severe defects in VD/DD dorsal axon outgrowth not significantly different from the *unc-6(ev400)* null (Table 2 and Fig. 3C). Likewise, *unc-6(zd146)* displayed strong ventral AVM axon guidance defects not significantly different from the *unc-6(ev400)* null (Table 2). These results indicate that *unc-6(zd146)*, a G289D missense mutation in the LN domain, is a strong loss-of-function mutation, comparable to a null. In the UNC-6 crystal structure, G289 makes two hydrogen bonds with I52 in a neighboring β-strand near the base of the ‘jelly roll’ β-barrel core of the LN domain (Fig. 2D). While the peptide backbone of the G residue is involved in the hydrogen bonding, insertion of a larger D residue might disrupt this interaction.

*unc-6(zd17)* mutants had less severe uncoordination compared to *unc-6(ev400)* or *unc-6(zd146)* mutants. Dorsal VD/DD axon guidance was only mildly affected in *unc-6(zd17)*, with an average of 15.82 axons emanating from the VNC and an average of 9.94 reaching the DNC, significantly less severe than the *unc-6(ev400)* null (Table 2 and Fig. 3D). However, ventral AVM axon guidance failed in 30% of *unc-6(zd17)* mutants (Table 2). Our data show that *unc-6(zd17)* strongly perturbed ventral axon guidance yet only weakly perturbed dorsal axon guidance.

The *zd17* mutation altered S120, a residue conserved in the LN domains of Netrins and laminins. In the UNC-6 crystal structure, S120 forms three hydrogen bonds with Y280 in an adjacent β-strand between the β-barrel ‘jelly roll’ core of the LN domain and a nearby domain that is potentially subject to glycosylation (Fig. 2E). Two of the three hydrogen bonds involve the serine side group. Insertion of a larger tyrosine residue likely disrupts these interactions.

### Dorsal-ventral axon guidance defects in EGF domain mutants

The cysteines affected by *unc-6(zd154)*, *unc-6(zd116)*, and *unc-6(e78)* are each involved in disulfide bonds with cysteines in the core structure of the three EGF domains (Fig. 2A-C). These mutations might predominantly affect the EGF domain in which they reside. Previous phenotypic analysis indicates that *unc-6(e78)* strongly affected dorsal axon guidance, with weaker effects on ventral axon guidance. For example, very few VD/DD commissures reached the DNC (only three in 100 animals scored), a proportion that is not significantly different from the *unc-6(ev400)* null, and only 4.54 on average emanated from the VNC, significantly fewer than in the *unc-6(ev400)* null (Table 2 and Fig. 3G). However, only 8% of AVM axons failed to extend ventrally, significantly fewer than the 72% observed for *unc-6(ev400)* and the 30% observed for *unc-6(zd17)* (Table 2). These results show that the EGF3 domain is required primarily for dorsal growth away from UNC-6 and support the idea that the *unc-6(zd17)* mutation might primarily affect ventral growth.

*unc-6(zd154)* affects a conserved cysteine in the first EGF domain. *unc-6(zd154)* affected both dorsal and ventral growth. *unc-6(zd154)* displayed on average 6.02 VD/DD axons emanating from the VNC, similar to the *unc-6(ev400)* null, and did not differ significantly from the *unc-6(ev400)* null for the number of axons reaching the DNC (Table 2 and Fig. 3E). *unc-6(zd154)* had significantly weaker effects on ventral AVM guidance compared to the *unc-6(ev400)* null, with 35% of AVM axons failing to extend ventrally in *unc-6(zd154)* (Table 2).

*unc-6(zd116)* alters a conserved cysteine in the second EGF domain, the analogous cysteine affected by *unc-6(e78)* in the third EGF domain. *unc-6(zd116)* affected both dorsal and ventral growth but was significantly weaker than the *unc-6(ev400)* null and the *unc-6(zd154)* EGF1 mutant (Table 2 and Fig. 3G). Furthermore, 18% of AVM axons failed to grow ventrally in *unc-6(zd116)* (Table 2), significantly weaker than the *unc-6(ev400)*. These data indicate that EGF domains 1 and 2 are required for both dorsal and ventral axon guidance, although perturbation of EGF1 more strongly affected dorsal axon guidance than EGF2. Consistent with previous results, EGF3 primarily affected dorsal axon guidance.

### A deletion in the Netrin C-terminal domain resembled a strong *unc-6* loss of function

*unc-6(zd144)* harbors a 132 bp deletion in a region encoding the Netrin C-terminal domain (Fig. 1 and Table 1). Previous studies indicate that the Netrin C-terminal domain is dispensable for D-V axon pathfinding and instead prevents ectopic axon branching (Wang and Wadsworth, 2002). *unc-6(zd144)* displayed strong defects in VD/DD dorsal axon pathfinding and AVM ventral axon pathfinding, although it is significantly weaker than the *unc-6(ev400)* null (Table 2). The *zd144* deletion includes the exon 11/intron 11 boundary (the third to the last exon) (Fig. 1 and Table 1). Possibly, splicing is disrupted in this mutant, resulting in a premature stop codon and nonsense-mediated transcript decay and causing the *unc-6* loss-of-function phenotype.

### Mutations in predicted N-glycosylation sites in UNC-6

UNC-6 contains three predicted N-glycosylation sites that are conserved in vertebrate Netrin-1 (Xu et al., 2014). The sites are N114 and N128 in the LN domain (Figs 1B and 2D,E) and N423 in the third EGF domain (Fig. 1B). N114 and N128 are glycosylated in the UNC-6 crystal structure, but N423 is not (Priest et al., 2024). We used Cas9 genome editing to convert these residues to alanine to eliminate potential glycosylation. *unc-6(lq194)* was N114A, *unc-6(lq196)* was N128A, and *unc-6(lq200)* was N423A. Additionally, *unc-6(lq194lq197)* was an N114A-N128A double mutant, and *unc-6(lq194lq197lq199)* was an N114A-N128A-N423A triple mutant. All three single mutants and these multiply mutant strains did not display apparent Unc defects. Only the *unc-6(lq194lq197lq199)* triple mutant had significantly more severe VD/DD defects compared to the wild type (15.58 versus 15.95 axons reaching the DNC) (Table 2). None of the mutants displayed ventral AVM axon guidance defects (Table 2). Our results reveal that even the loss of all three predicted N-glycosylation sites only had weak effects on dorsal-ventral axon guidance.

### *unc-6 N114*, *N128*, and *N423* mutations interact genetically with *unc-40* and *unc-5*

UNC-40/DCC acts as a receptor with UNC-6 in both dorsal and ventral axon guidance (Norris and Lundquist, 2011). The dorsal axon guidance defects in *unc-40(n324)*, a strong loss-of-function mutant, were weaker than those in *unc-6(ev400)*, with an average of 15.76 axons emerging from the ventral cord and an average of 9.88 reaching the DNC (Table 2 and Fig. 3H), similar to what has been previously reported (Norris and Lundquist, 2011). In double mutants with *unc-40(n324)*, each asparagine-altered mutant significantly enhanced defective growth to the DNC but not the extension of axons emerging from the VNC (Table 2 and Fig. 3I).

UNC-5 acts as a receptor with UNC-6 in primarily dorsal axon guidance. In *unc-5* null mutants, very few VD/DD axons emerge from the ventral cord (Table 2) (Gujar et al., 2018). *unc-5(ev480)* is a hypomorphic mutation affecting only the full-length *unc-5A* isoform (Mahadik and Lundquist, 2023). *unc-5(ev480)* mutants displayed an average of 10.72 VD/DD axons emerging from the VNC and an average of 6.61 reaching the DNC (Table 2). The *unc-6(lq194lq197lq199)* triple mutant significantly enhanced VD/DD defects of *unc-5(ev480)* (Table 2). Each of the single mutants enhanced defects in reaching the dorsal cord (Table 2). Surprisingly, each single mutant and the double mutant significantly suppressed defective emergence from the VNC of *unc-5(ev480)* (Table 2), suggesting a complex genetic interaction with *unc-5*.

*unc-40(n324)* displayed 11% failure of ventral AVM axon guidance (Table 2 and Fig. 4C). *unc-6(lq194lq197lq200)* did not significantly enhance ventral AVM axon guidance defects in *unc-40(n324)* (Table 2). The strong loss-of-function *unc-5(e791)* mutants displayed 4% ventral AVM axon guidance defects, whereas the hypomorphic *unc-5(ev480)* displayed no defects (Table 2). The single, double and triple mutants with *unc-5(ev480)* each displayed weak ventral AVM axon guidance defects, but they did not differ significantly from *unc-5(ev480)* alone. The *unc-6(lq194lq197lq200)* triple mutant did not significantly enhance the strong *unc-5(e791)* mutant (Table 2).

N114 and N128 both have carbohydrate linkages in the crystal structure (Fig. 5A,B), but N423 does not (Fig. 5C). N114 and N128 make no other hydrogen bonds with surrounding residues, but N423 in EGF3 makes three hydrogen bonds (Fig. 5). Thus, N423 might weakly perturb the structure of EGF3 in addition to blocking potential N-glycosylation.

**Fig. 5.**
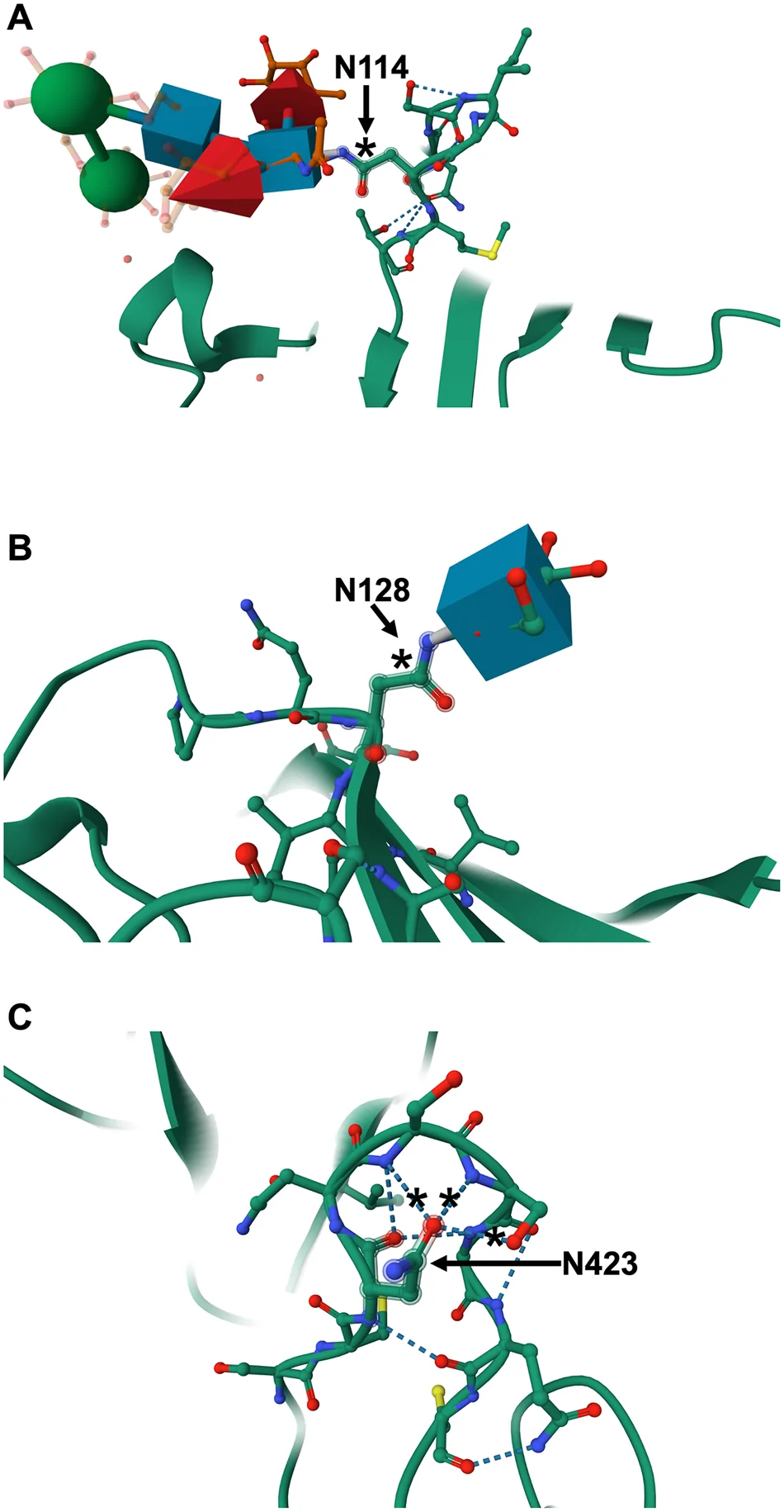
**Potential glycosylation sites in UNC-6.** See Fig. 2 for structure generation. Three conserved potential N-glycosylation sites were found in UNC-6. (A) In the UNC-6 crystal structure, N114 has a complex alpha-D-mannopyranose linkage (blue boxes, red cones, and green spheres). (B) N128 has a 2-acetomido-2-deoxy-beta-D-glucopyranose linkage (blue box). N114 and N128 do not make other hydrogen bonds. (C) N423 has no carbohydrate linkage and instead makes three hydrogen bonds with neighboring residues in the EGF3 domain (asterisks).

## DISCUSSION

### Mutations in the LN domain

Four of the new *unc-6* mutations are missense mutations that alter distinct domains of UNC-6. Previously described mutations in the *unc-6* LN domains perturbed both dorsal and ventral growth (Lim and Wadsworth, 2002; Xu et al., 2009). *zd17* (S120F) and *zd146* (G289D) in the LN domain had distinct effects on dorsal-ventral axon pathfinding. Dorsal VD/DD axon guidance defects and ventral AVM axon guidance defects in *unc-6(zd146)* were comparable to the null *unc-6(ev400)*, indicating that the conserved glycine 289 is needed for all UNC-6 functions. The importance of this residue is highlighted by the mutation of the residue in laminin α2, causing limb-girdle-type dystrophy in humans (Gavassini et al., 2011), and in laminin β1, causing cardiac development defects in *Drosophila* (Hollfelder et al., 2014). By contrast, *unc-6(zd17)* was a hypomorphic *unc-6* mutation that more strongly disrupted ventral axon pathfinding than dorsal. S120 makes hydrogen bonds with an adjacent β-strand in a domain outside of the ‘jellyroll domain’ that is glycosylated at N114 (Fig. 2E). However, mutation of N114 did not disrupt dorsal or ventral axon guidance, arguing that another aspect of the S120F mutation is perturbing ventral axon guidance. A distinct region of the LN domain of Netrin-1 is required for interactions with the fourth fibronectin domain of DCC and Neogenin (Xu et al., 2014), potentially explaining the role of the LN domain in both dorsal and ventral axon guidance. The S120F mutation in *zd17* might specifically disrupt interactions with UNC-40 or another receptor affecting ventral but not dorsal axon guidance. LN domains also interact with one another, forming the laminin lattice network in the basal lamina (Shaw et al., 2021; Kulczyk et al., 2023). *zd17* might affect the interaction of UNC-6 with laminin, which might be required for ventral axon guidance. A caveat to this domain-specific interpretation of these results is that the missense mutations might generally perturb protein stability and/or levels, which results in the distinct phenotypes we document.

### Mutations of the three EGF domains

EGF3 is specifically involved in dorsal axon guidance (Lim and Wadsworth, 2002; Norris and Lundquist, 2011). We found that *unc-6(e78)*, which alters a disulfide-bond-forming cysteine residue in EGF3 (Fig. 2C), had strong defects in dorsal VD/DD axon guidance but did not strongly affect ventral AVM growth (Table 2), consistent with these results. EGF3 of Netrin-1 interacts physically with the fifth fibronectin domain of DCC (Xu et al., 2014). In *C. elegans*, UNC-40 is involved in both dorsal and ventral growth (Norris and Lundquist, 2011; Norris et al., 2014), so it is possible that potential EGF3 interactions with UNC-40 are specific for dorsal growth.

EGF2 is required for both dorsal and ventral growth (Lim and Wadsworth, 2002). *unc-6(zd116)* altered a disulfide-forming cysteine in EGF2 (Fig. 2B). *unc-6(zd116)* mutants had defects in dorsal VD/DD and ventral AVM axon guidance that were significantly less penetrant than those for the *unc-6(ev400)* null. Our data indicate that EGF2 is required for both dorsal and ventral axon guidance, consistent with previous structure-function studies.

*unc-6(zd154)* affects a disulfide-forming cysteine in EGF1 (Fig. 2A), and mutants had strong defects in dorsal VD/DD axon guidance similar to the *unc-6(ev400)* null. Effects on ventral AVM axon guidance were weaker than *unc-6(ev400)*. Our data show that EGF2 is involved in both dorsal and ventral axon guidance, but with a more pronounced role in dorsal axon guidance. This observation is consistent with AlphaFold predictions of the UNC-5 second immunoglobulin domain interacting with EGF1 (Priest et al., 2024), possibly to mediate dorsal growth away from UNC-6.

In sum, our analysis of domain-specific missense mutations suggests that the LN domain, EGF1, and EGF2 are required for both dorsal VD/DD and ventral AVM axon guidance to varying extents and that EGF3 is required for dorsal VD/DD axon guidance with a minimal role in ventral axon guidance (Fig. 6). The S120F region in the LN domain might predominantly be required for ventral AVM axon guidance. One caveat is that domain-specific mutations might have secondary effects on UNC-6 overall structure and stability, making it difficult to assign specific functions to specific domains. However, previous structure-function studies (Lim and Wadsworth, 2002; Xu et al., 2009) and physical interaction studies (Xu et al., 2014; Priest et al., 2024) are consistent with domain-specific functions.

**Fig. 6.**
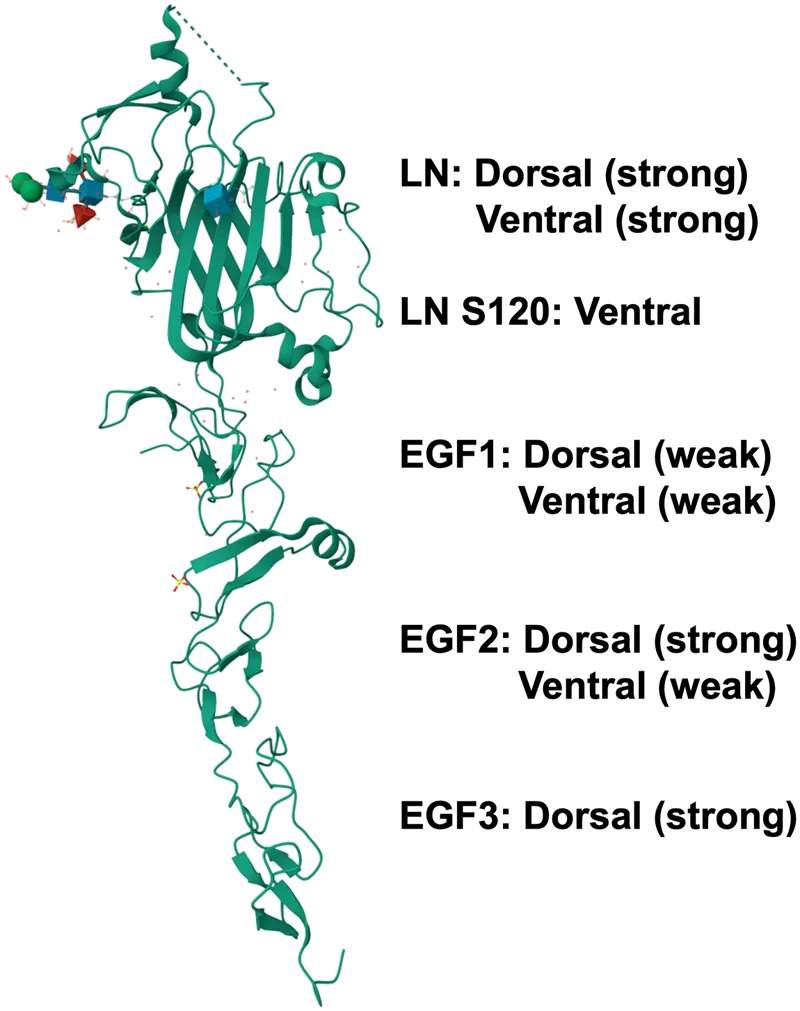
**Roles of UNC-6 regions in dorsal VD/DD and ventral AVM axon guidance.** See Fig. 2 for structure generation. Disruption of the LN domain strongly perturbed both dorsal VD/DD and ventral AVM axon guidance, and alteration of the S120 residue in the LN domain predominantly perturbed ventral AVM axon guidance. Mutation of the EGF1 domain perturbed both dorsal and ventral axon guidance but with weaker effects on ventral axon guidance. Disruption of the EGF2 domain weakly perturbed both dorsal and ventral axon guidance. Mutation of EGF3 predominantly disrupted dorsal VD/DD axon guidance.

### Potential N-glycosylation site mutations interacted genetically with *unc-40* and *unc-5*

UNC-6 N114, N128, and N423 are predicted N-glycosylation sites. Indeed, N114 and N128 had carbohydrate linkages in a crystal structure, whereas N423 did not (Priest et al., 2024) (Fig. 2). Mutations altering each individual site to alanine had no detectable effect on dorsal-ventral axon guidance, nor did double or triple mutants. However, N114, N128, and N114-N128 enhanced the dorsal VD/DD axon guidance defects of *unc-40* mutants. Possibly, these sites function in parallel to UNC-40 in dorsal axon guidance.

Surprisingly, the single mutants and the N114A-N128A double mutant suppressed the hypomorphic *unc-5(ev480)* mutation for the number of axons emanating from the VNC. This observation might reflect the balance of UNC-5 and UNC-40 responses to UNC-6 in dorsal VD/DD outgrowth in the polarity/protrusion model (Norris and Lundquist, 2011; Gujar et al., 2018). In the absence of UNC-5 repulsive signaling, UNC-6 might draw the axons back to the VNC via attractive signaling and UNC-40 (Norris and Lundquist, 2011; Norris et al., 2014; Gujar et al., 2017, 2018, 2019; Mahadik and Lundquist, 2023; Hooper et al., 2025). This explains why the dorsal VD/DD defects of *unc-5* mutants are stronger than those of *unc-6* and are suppressed by *unc-40* (Norris and Lundquist, 2011). Possibly, UNC-6 function is slightly perturbed in N114A, N128A, and N423A, resulting in reduced UNC-5 signaling and less growth back toward the VNC. *unc-5(ev480)* with the triple mutant N114A-N128A-N423 showed significantly fewer VD/DD axons emerging from the VNC, indicating that the cumulative effect of the three mutants begins to have more severe loss of *unc-6* function, which enhances *unc-5(ev480)*. The number of VD/DD axons reaching the VNC in *unc-5(ev480)* was significantly reduced by each single mutant and the double and triple mutants. Possibly, once the axons have left the VNC, the mutation UNC-6 acts with UNC-5, and the N114A, N128A, and N423A mutations enhance the phenotype of the *unc-5(ev480)* hypomorph.

For ventral AVM axon guidance, the triple N114A-N128A-N423A mutant did not significantly enhance *unc-40(n324)* (Table 2). However, the mutations did cause ventral AVM axon guidance in the context of the *unc-5(ev480)* hypomorph to an extent similar to the *unc-5(e791)* null. For example, *unc-5(ev480)* showed no ventral AVM axon guidance defects, whereas the *unc-5(ev480); N114AN128An423A* mutants showed 7% defects, similar to *unc-5(e791)*. Together, these data indicate that N114, N128A, and N423 slightly perturb the UNC-6 function revealed in the context of genetic interactions with *unc-40* and *unc-5*. Biochemical and structural analyses reveal that UNC-5 requires heparin for strong binding to UNC-6, which also excludes UNC-40 interaction (Priest et al., 2024). The relation of this result to N114A and N128A is unclear, but it is possible that glycosylation of UNC-6 at these residues is required for robust interaction with UNC-5. It is also possible that these mutations each slightly perturb the structure or stability of UNC-6 in a cumulative fashion. In any case, it is unlikely that N423 is a *bona fide* glycosylation site and instead might be involved in hydrogen bonding in the EGF3 domain structure (Priest et al., 2024) (Fig. 5C).

In sum, these studies delineate the roles of specific domains of UNC-6/Netrin in dorsal VD/DD axon guidance and ventral AVM axon guidance using missense mutations in the endogenous *unc-6* locus. The LN domain was required for both dorsal and ventral axon guidance, but S120 in the LN domain was specific to ventral AVM axon guidance. EGF1 and EGF2 were required for both dorsal and ventral axon guidance, and EGF3 was specific to dorsal axon guidance as previously reported. Finally, N-linked glycosylation at N114 and N128 was not required for UNC-6 function in a *wild-type* background but modified dorsal and ventral axon guidance in *unc-40* and *unc-5* mutant backgrounds. Our results will inform future studies of how these distinct domains interact with the guidance receptors UNC-40 and UNC-5 and other extracellular molecules to control dorsal-ventral axon guidance.

## MATERIALS AND METHODS

### Genetics

LG I: *unc-40(n324)*, *zdIs5[Pmec-4::gfp]*. LG II: *juIs76[Punc-25::gfp]*. LG IV: *unc-5(e791 and ev480)*, *zdIs4[Pmec-4;;gfp]*. LG X: unc-6(ev400, e78, zd6, zd17, zd21, zd59, zd61, zd81, zd116, zd121, zd123, zd131, zd144, zd146, zd154, zd156, lq194, lq196, lq200, lq194lq197, and lq194lq197lq199). LG IV: zdIs3[Pglr-1::gfp]. LG V: oyIs14[Psra-6::gfp] V. lqEx1241[unc-6(+)] is an extrachromosomal array.

To identify axon guidance mutants, we treated animals containing neuron-specific GFP reporters (*oyIs14[Psra-6::gfp] V, zdIs3[Pglr-1::gfp] IV*) with the mutagen ethyl methanesulfonate (EMS) (Brenner, 1974) or ethyl nitrosourea (ENU) (De Stasio and Dorman, 2001) and then screened their F2 progeny using fluorescence microscopy for animals with altered axon trajectories. Eleven *unc-6* alleles were recovered in screens using *oyIs14[Psra-6::gfp]* following EMS (*zd6*, *zd17*, *zd81*, *zd116*, *zd121*) and ENU (*zd123*, *zd131*, *zd144*, *zd146*, *zd154*, *zd156*) treatments. Three *unc-6* alleles were isolated in screens using *zdIs3[glr-1::gfp]* following EMS (*zd21*, *zd59*, *zd61*) treatment. The 14 new *unc-6* alleles were tested for complementation of the null *unc-6(ev400)* allele using the Unc phenotype. *lqEx1241[Punc-6::unc-6(+)]* is a transgene that rescues the *unc-6* Unc phenotype and axon guidance defects (Hooper et al., 2025). The *juIs76[Punc-25::gfp]* transgene (Jin et al., 1999) was used to visualize the VD and DD GABAergic motor neurons (Hooper et al., 2025). *unc-6(ev400) X; juIs76 II; lqEx1241* males were mated to each of the 14 *unc-6* alleles. Cross progeny containing the *juIs76* transgene (e.g. *zd6/ev400 trans-*heterozygotes) were scored for the Unc phenotype. Those cross progeny that inherited the *lqEx1241* transgene will be non-Unc even in the case of non-complementation because of the rescuing transgene. Animals that did not inherit the *lqEx1241* transgene would not be rescued and would be Unc if the new allele failed to complement *unc-6(ev400)*. Both male and hermaphrodite cross progeny with *juIs76* were scored. For each of the 14 alleles, Unc cross progeny were detected, indicating failure to complement *unc-6(ev400)* for Unc.

To confirm that the mutations are alleles of *unc-6*, each of the new mutations was crossed with the *lqEx1241 unc-6(+)* rescuing transgene. *lqEx1241* males were mated to each of the 14 new mutations. In each case, we found non-Unc male cross progeny that were hemizygous for the new *unc-6* mutant and carried the *lqEx1241* rescuing transgene. These males were mated back into the new *unc-6* mutant, and non-Unc cross-progeny hermaphrodites were selected, which were homozygous for the new *unc-6* allele and harbored the *lqEx1241* rescuing transgene. These were used to create stable, homozygous rescued lines for each of the 14 new *unc-6* mutants. Mutant strains are available upon request from the corresponding author or at the Caenorhabditis Stock Center.

### *unc-6* allele sequencing

The *unc-6* locus from each of the 14 new *unc-6* alleles was amplified by PCR in two overlapping fragments (see Supplemental Materials for primer sequences). The amplified fragments were subject to Oxford Nanopore DNA sequencing (Plasmidsaurus, USA). FASTQ reads were aligned to the genome using BWA-MEM2 (Li and Durbin, 2009). The resulting BAM files were analyzed on the Integrative Genomics Viewer (Robinson et al., 2011; Thorvaldsdottir et al., 2012). Sequence alterations in each of the 14 new *unc-6* strains were identified (Table 1). Primer sequences used for amplicon sequencing are in Supplemental File 1.

### Scoring dorsal-ventral axon guidance defects

L4 or young adult hermaphrodite *C. elegans* were scored. The *juIs76[Punc-25::gfp]* (Jin et al., 1999) transgene was used to score VD/DD GABAergic dorsal axon guidance (Hooper et al., 2025). The 19 VD and DD axons grow from the VNC to the DNC to form commissures. In wild type, an average of 16 commissures is observed due to some axons extending in the same commissural fascicle (Table 1 and Fig. 3A). For each genotype, the number of axons emanating from the VNC and the number of commissures reaching the DNC were counted (Table 1). 100 animals were scored. One-way ANOVA with Tukey's post hoc test was used to statistically compare genotypes and to correct for multiple testing (see Supplemental Materials for all raw data and analysis). ANOVA was used on three groups: *wild type* and *unc-6* mutants; *wild type*, *unc-40(n324)*, double mutants with *unc-40(n324)*; and *wild-type*, *unc-5* mutants, double mutants with *unc-5(ev480)*.

The *zdIs4* and *zdIs5 Pmec-4::gfp* transgenes (Royal et al., 2005; Hooper et al., 2025) were used to score ventral AVM axon guidance. In wild type, the AVM axon emanates from the cell body directly to the VNC and then extends anteriorly in the VNC (Fig. 4). For each genotype, the percentage of AVM axons failing to emanate directly to the ventral cord from the cell body was determined. In most cases, the defective axons extended anteriorly along the lateral region (Fig. 4). 100 AVM axons were scored. In *unc-40*, AVM was sometimes displaced posteriorly due to Q neuroblast and descendant migration defects (Honigberg and Kenyon, 2000; Sundararajan and Lundquist, 2012). Only AVMs in the *wild-type* position were scored in *unc-40* mutants. Fisher's exact test was used for pairwise statistical comparisons between genotypes.

### Cas9 genome editing

The potential glycosylation sites N114, N128, and N423 in *unc-6* were changed to alanine residues using Cas9 genome editing and standard techniques (Dickinson et al., 2013; Norris et al., 2015). See Supplemental Material for the sequences of synthetic guide RNA *unc-6* inserts, the single-stranded oligonucleotide repair templates, and the sequences of the genome-edited *unc-6* mutants. A mix of single-guide RNAs, a single-stranded oligonucleotide repair template, and the Cas9 enzyme was injected into the gonads of N2 animals, along with the *dpy-10(cn64)* co-CRISPR reagents (El Mouridi et al., 2017). Genome-edited animals were identified using PCR with primers specific to the recoded regions introduced from the repair template. The genome edits were confirmed by PCR and sequencing. Genome-editing reagents were produced by InVivo Biosystems (Eugene, OR, USA).

Individual *lq194 N114A*, *lq196 N128A*, and *lq200 N423A* were generated in a *wild-type* N2 background. *lq197 N128A* was induced in the *lq194 N114A* background to create the *lq194lq197* double mutant. This required the use of a novel repair template oligonucleotide that corresponded to the recoded sequence of the N114A genome edit. *lq199 N423A* was induced in the *lq194lq197* background to create the *lq194lq197lq199* triple mutant. Primer sequences for genome editing can be found in Supplemental File 1.

### Generation of crystal structure representations

Images in Figs 2 and 5 were generated by Mol* from the X-ray crystal structure of the UNC-6 LN and EGF domains in the Protein Data Bank (Berman et al., 2000; Priest et al., 2024) (pdb00008EDK).

## Supplementary Material



10.1242/biolopen.062822_sup1 Supplementary information
