# Supplementary material for: Domain-specific mutations in unc-6/Netrin differentially affect dorsal-ventral axon pathfinding in Caenorhabditis elegans
Source: Biol Open. 2026 Sep 16;15(9):bio062822. doi: 10.1242/bio.062822 (PMC13617976; doi:10.1242/bio.062822)
Supplement: Supplementary file 1 [file biolopen-15-062822-s1.pdf]

Table S1. Primer sequences used in this work

Name	Purpose	Sequence
unc-6amp1F	Amplicon sequencing of *unc-6* alleles	agtcaatttccagattgtgtc
unc-6amp1R	Amplicon sequencing of *unc-6* alleles	gtgctgactgaccagtaggta
unc-6amp2F	Amplicon sequencing of *unc-6* alleles	ggtggtagccattcttccgtg
unc-6amp2R	Amplicon sequencing of *unc-6* alleles	gaacagcttgatatggctgcc
N114AsgRNA	sgRNA insert for the N114A genome edit	cgattggaaacatgacatgctgg
N128AsgRNA	sgRNA insert for the N128A genome edit	ccaagtttgagcccacaaaacgt
N423AsgRNA	sgRNA insert for the N423A genome edit	aaaagctgcaaccaatcatcggg
N114AssODN	Repair template oligonucleotide for the N114A genome edit	gtgatgctagaaaccatttccaatcc catccagcctccctccttaccgacctt aattctatcggagctatgacttgttgg gtctctactccaagtttgagcccacaa aacgtttcactcactttg
N128AssODN	Repair template oligonucleotide for the N128A genome edit	ttcgattggaaacatgacatgctgggttt ccactccgtccctttctccacaggccgtc tcccttaccctttcccttggaaagaaattc gaacttacctacgtttccatgcatttttgct ctagactcccagattcaatggcactttac aagtctgctgac
N114N128AssODN	Repair template oligonucleotide for the N128A genome edit in the N114A background	ttctatcggagctatgacttgttgggtc tctactccgtccctttctccacaggcc gtctcccttaccctttcccttggaaag aaattcgaacttacctacgtttccatg catttttgctctagactcccagattcaa tggcactttacaagtctgctgact
N423AssODN	Repair template oligonucleotide for the N423A genome edit	tcaaaaccacttaagcttgtggatgtc atccagtcggtagcctcggtaagtctt gtgctcagtcatcgggtcagtgcgtct gcaagcctggagtcactg

Biology Open • Supplementary information
