# Supplementary material for: Domain-specific mutations in unc-6/Netrin differentially affect dorsal-ventral axon pathfinding in Caenorhabditis elegans
Source: Biol Open. 2026 Sep 16;15(9):bio062822. doi: 10.1242/bio.062822 (PMC13617976; doi:10.1242/bio.062822)
Supplement: Supplementary file 2 [file biolopen-15-062822_review_history.pdf]

# Domain-specific mutations in unc-6/Netrin differentially affect dorsal-ventral axon pathfinding in Caenorhabditis elegans

Kelsey Hooper, Scott Clark and Erik Lundquist
10.1242/bio.062822

**Editor**: Sandhya Koushika

**Review timeline**

| | |
|---|---|
| Original submission: | 16 July 2026 |
| Editorial decision: | 28 July 2026 |
| First revision received: | 18 August 2026 |
| Accepted: | 25 August 2026 |

---

**Original submission**

First decision letter

MS ID#: bio.062822

MS Title: Domain-specific mutations in unc-6/Netrin differentially affect dorsal-ventral axon pathfinding in Caenorhabditis elegans

Authors: Kelsey Hooper; Scott Clark; Erik Lundquist

I have now reached a decision on the above manuscript.

The reviewer reports are shown at the bottom of this email.

**As you will see, the reviewers raised a number of substantial criticisms that prevent me from accepting the paper at this stage.**

They suggest, however, that a revised version might prove acceptable, if you can address their concerns. If you think that you can deal satisfactorily with the criticisms on revision, I would be pleased to see a revised manuscript. We would then return it to the reviewers.

At this stage, we also ask you to ensure your manuscript complies with our formatting guidelines. Provided you are able to fully address the referees' comments, we are positive about publication of your paper (we accept over 95% of revision submissions) and therefore hope you won't mind any extra work involved in reformatting your manuscript at this point.

Please upload both a 'clean' version of your Word file, along with a highlighted version clearly showing where you have made changes in the revised manuscript. Please avoid using 'Track changes' in Word files as these are lost in PDF conversion.

I should be grateful if you would also provide a point-by-point response detailing how you have dealt with the points raised by the reviewers in the 'Response to Reviewers' box. Please attend to all of the reviewers' comments. If you do not agree with any of their criticisms or suggestions please explain clearly why this is so.

© 2026. Published by The Company of Biologists under the terms of the Creative Commons Attribution License (https://creativecommons.org/licenses/by/4.0/).

Reviewer 1

Comments for the author

The study presents a structure-function analysis of the UNC-6/Netrin protein through the characterization of fourteen new alleles, including four missense mutations. By examining these mutations in the endogenous locus, the authors analyze the contribution of different protein domains to axon guidance. In the LN domain, the conserved G289D substitution behaves as a strong loss-of-function allele and affects both dorsal and ventral guidance. In contrast, the S120F substitution produces a partial defect that primarily affects ventral AVM axon guidance while having little effect on dorsal guidance, indicating that this region contributes to ventral attraction. Mutations in the EGF domains show distinct phenotypes: substitutions in EGF1 (C321G) and EGF2 (C347Y) affect both dorsal and ventral guidance to different extents, whereas the EGF3 mutation (C410Y) specifically alters dorsal VD/DD guidance, consistent with previous transgenic studies. The authors also used Cas9 genome editing to remove the predicted N-glycosylation sites N114 and N128. These modifications did not produce guidance defects on their own but showed genetic interactions with the UNC-40/DCC and UNC-5 receptors, indicating that these sites contribute to signaling in combination with receptor function.

The use of missense mutations in the endogenous unc-6 locus provides an alternative to previous analyses based on domain-deletion transgenes and allows the effects of individual amino acid substitutions to be examined in their native genomic context. The structural interpretation of the mutations is supported by recently available X-ray crystallography data, including the work of Priest et al. (2024), which is used to relate individual substitutions to potential disruption of hydrogen bonds or disulfide bridges. The results are also consistent with previous studies describing the functional roles of Netrin domains, including Lim and Wadsworth (2002) and Norris and Lundquist (2011).

One limitation is that some domain-specific mutations may alter protein stability rather than directly affecting domain-specific activity. For example, the zd144 deletion could result in nonsense-mediated decay of the transcript, making it difficult to distinguish between loss of protein expression and disruption of a specific functional domain. In addition, although the genetic analysis identifies hypomorphic alleles such as S120F, direct measurements of protein abundance or localization, for example by Western blotting or immunohistochemistry, would help determine whether these proteins are expressed at normal levels.

The statistical analyses are appropriate for the experimental design. One-way ANOVA followed by Tukey's post hoc test was used for the analysis of VD/DD motor neuron guidance across multiple genotypes, and Fisher's exact test was applied to pairwise comparisons of AVM ventral guidance defects. The primary guidance assays included scoring of 100 animals per genotype.

The information provided allows the experiments to be reproduced. The manuscript includes descriptions of the alleles, sequence changes, and genetic backgrounds, primer sequences for amplicon sequencing and CRISPR repair templates, and publicly available RNA-seq FASTQ files. The experimental data support the conclusions regarding the differential effects of the S120F and EGF3 mutations on ventral and dorsal guidance, respectively. The genetic interaction analysis of the glycosylation-site mutants also supports the conclusion that these residues are not individually required for guidance but contribute to receptor-dependent signaling.

The methods are described in sufficient detail. The combination of EMS and ENU mutagenesis enabled the recovery of multiple allele classes. Nanopore amplicon sequencing was used to identify mutations generated in the genetic screens, and the Cas9 genome-editing procedures follow established protocols. The GFP markers juIs76 and zdIs5 were used to visualize the corresponding axons.

Several minor editorial issues should be corrected. The phrase "partially disturpted AVM ventral guidance" should be changed to "partially disrupted AVM ventral guidance." The sentence beginning with "the zd144 deletion includes..." should begin with a capital letter. The capitalization of "Cas9" should be used consistently throughout the manuscript. The phrase "...detectable effect of dorsal-

© 2026. Published by The Company of Biologists under the terms of the Creative Commons Attribution License (https://creativecommons.org/licenses/by/4.0/). 

ventral axon guidance..." should likely read "...detectable effect on dorsal-ventral axon guidance..."

Figure labels describing the three-dimensional protein structures should be formatted consistently across all panels. Citations in the text, including Hooper et al. (2025), should be checked against the final bibliography.

Reviewer 2

Comments for the author

This manuscript reports the isolation and characterization of fourteen new unc-6/Netrin alleles identified through genetic screens in C. elegans. The authors identify missense mutations affecting the LN, EGF1, EGF2, and EGF3 domains of UNC-6 and analyze their effects on dorsal VD/DD and ventral AVM axon guidance. In addition, CRISPR-generated mutations at three predicted N-glycosylation sites are examined alone and in sensitized genetic backgrounds.

The study represents a wide genetic structure-function analysis of UNC-6 using endogenous alleles rather than transgenic deletion constructs. The recovery of multiple independent alleles, complementation tests, rescue experiments, molecular characterization, and quantitative phenotypic analysis provide strong support for the conclusions regarding domain-specific contributions to axon guidance.

Overall, the manuscript is technically good and provides useful insights into UNC-6 domain function. However, several aspects of data presentation and interpretation could be improved.

Major comments:

1. Glycosylation-related conclusions should be moderated:

Some statements regarding N-glycosylation appear stronger than what is showed by the data. The genetic results clearly demonstrate functional consequences of mutating conserved residues that may be glycosylated. However, effects on glycosylation itself remain inferred. I recommend revising the text to distinguish between mutation of glycosylation acceptor residues, and direct evidence for glycosylation-dependent regulation. No additional experiments are necessarily required, but the interpretation should be more cautious.

Examples:

-Line 44 in the abstract: "…these glycosylation sites indeed have a role in UNC-6 signaling" consider "… that these conserved residues contribute to UNC-6 signaling."

-Line 106 in the intro "To test the role of N-glycosylation…". Glycosylation itself was not tested

-Line 110 in the intro. "glycosylation sites were eliminated", I suggest claiming "...UNC-6 function was modestly reduced when these conserved asparagine residues were mutated." Similar in final discussion paragraph starting in line 478.

2. Data presentation:

The valuable data present by the manuscript could benefit of a better presentation. The reading needs an uncomfortable movement cross referencing along tables and figures for each result fragment. I would suggest considering a more integrated graphical presentation like a domain-level summary figure combining (i) mutation location, (ii) phenotype severity, and (iii) affected guidance modality.

Table 2 includes some p values express as "p < 0.0001" and others with the exact value "p = 0.0100". please clarify or homogenize this. Consider reporting: 1) exact p-values where possible rather than simply "NS", 2) measures of variance in phenotype tables and 3) graphical presentation of animal-

© 2026. Published by The Company of Biologists under the terms of the Creative Commons Attribution License (https://creativecommons.org/licenses/by/4.0/).

level distributions. Although the sample sizes are appropriate, visualization of data distributions would make interpretation easier.

Structural figures occupy substantial space relative to their role in the manuscript: Figures 2 and 5 are useful for visualizing residue locations and possible structural consequences but these panels are based on previously published structures rather than structural analyses performed in the current study. As the manuscript's primary contribution is genetic and phenotypic rather than structural. The authors may wish to consider whether some structural panels could be condensed or moved to supplementary material. Also, this figures generation are missing in the "Methods". The manuscript would benefit of clarifying how this was generated and visualized.

Minor comments:

3. Number of genomes screened

The manuscript would benefit from reporting the approximate scale of the EMS/ENU screens (number of genomes screened, number of F2 animals examined, and total number of axon-guidance mutants recovered). This information would provide useful context regarding screen saturation and the frequency with which unc-6 alleles were recovered.

4. Glycosylation sites. N423

The manuscript discusses N423 alongside N114 and N128, yet later notes (line 279) that N423 is unlikely to represent a bona fide glycosylation site. This distinction should be emphasized earlier to avoid confusion.

Typos and small comments to address:

- Several acronyms are not clarified the first time written: AVM, VD, DD, HSN neuron

- Line 53, "reviewed in…". Just add as one more reference.

- Line 39, in the abstract. Potential typo: "S120Y". is it "S120F"?

- line 109 extra space after mutants

- line 147 -150 different typography

- line 230: "the" ◊ The. Caps missing

- line 232 and 232 "resulting" is repeated

- line 238, reference missing on Netrin 1 in vertebrates' statement.

- line 256, again, "previously reported". Clarify where

- line 390 and line 418 use second and forth

- Clarification on line 13 on Scott G. Clark affiliation "(retired)". Check with journal how to state this.

Reviewer's Responses to Questions

**Experimental quality**

Are experiments performed using appropriate methods that will answer the question (or test the hypothesis or support the observations) posed by the authors? Is the right tool used for the job?
*If 'No', please indicate reasons in Comments for Author box below.*
Reviewer #1:

© 2026. Published by The Company of Biologists under the terms of the Creative Commons Attribution License (https://creativecommons.org/licenses/by/4.0/).

- Yes

Reviewer #2:
- Yes

---

Were the data analyzed using appropriate statistical tests?
*If 'No', please indicate reasons in Comments for Author box below.*
Reviewer #1:
- Yes

Reviewer #2:
- Yes

---

**Reproducibility**
Were experiments performed using adequate number of biological replicates?
*If 'No', please indicate reasons in Comments for Author box below.*
Reviewer #1:
- Yes

Reviewer #2:
- Yes

---

Is there sufficient raw data to assess the rigor of the analysis?
*If 'No', please indicate reasons in Comments for Author box below.*
Reviewer #1:
- Yes

Reviewer #2:
- Yes

---

Does the methods section provide sufficient detail to permit reproducibility?
*If 'No', please indicate reasons in Comments for Author box below.*
Reviewer #1:
- Yes

Reviewer #2:
- Yes

---

**Completeness**

Are the manuscript's conclusions supported by the data?
*If 'No', please indicate reasons in Comments for Author box below.*
Reviewer #1:
- No

Reviewer #2:
- Yes

---

**Scholarship**

Do the authors cite and discuss the merits of data that would argue for and against their conclusion?
*If 'No', please indicate reasons in Comments for Author box below.*

© 2026. Published by The Company of Biologists under the terms of the Creative Commons Attribution License (https://creativecommons.org/licenses/by/4.0/).

Reviewer #1:

- Yes

Reviewer #2:

- Yes

---

Does the manuscript title & abstract accurately reflect the contents of the manuscript, without hyperbole?
*If 'No', please indicate reasons in Comments for Author box below.*
Reviewer #1:

- Yes

Reviewer #2:

- Yes

---

**First revision**

Author response to reviewers' comments

Responses in BLUE

Reviewer 1: The study presents a structure-function analysis of the UNC-6/Netrin protein through the characterization of fourteen new alleles, including four missense mutations. By examining these mutations in the endogenous locus, the authors analyze the contribution of different protein domains to axon guidance. In the LN domain, the conserved G289D substitution behaves as a strong loss-of-function allele and affects both dorsal and ventral guidance. In contrast, the S120F substitution produces a partial defect that primarily affects ventral AVM axon guidance while having little effect on dorsal guidance, indicating that this region contributes to ventral attraction. Mutations in the EGF domains show distinct phenotypes: substitutions in EGF1 (C321G) and EGF2 (C347Y) affect both dorsal and ventral guidance to different extents, whereas the EGF3 mutation (C410Y) specifically alters dorsal VD/DD guidance, consistent with previous transgenic studies. The authors also used Cas9 genome editing to remove the predicted N-glycosylation sites N114 and N128. These modifications did not produce guidance defects on their own but showed genetic interactions with the UNC-40/DCC and UNC-5 receptors, indicating that these sites contribute to signaling in combination with receptor function.

The use of missense mutations in the endogenous unc-6 locus provides an alternative to previous analyses based on domain-deletion transgenes and allows the effects of individual amino acid substitutions to be examined in their native genomic context. The structural interpretation of the mutations is supported by recently available X-ray crystallography data, including the work of Priest et al. (2024), which is used to relate individual substitutions to potential disruption of hydrogen bonds or disulfide bridges. The results are also consistent with previous studies describing the functional roles of Netrin domains, including Lim and Wadsworth (2002) and Norris and Lundquist (2011).

One limitation is that some domain-specific mutations may alter protein stability rather than directly affecting domain-specific activity. For example, the zd144 deletion could result in nonsense-mediated decay of the transcript, making it difficult to distinguish between loss of protein expression and disruption of a specific functional domain. In addition, although the genetic analysis identifies hypomorphic alleles such as S120F, direct measurements of protein abundance or localization, for example by Western blotting or immunohistochemistry, would help determine whether these proteins are expressed at normal levels.

We agree that the missense mutations could be affecting protein stability and/or levels, which results in the specific effects observed. We have added a statement raising this possibility as an alternate explanation:

© 2026. Published by The Company of Biologists under the terms of the Creative Commons Attribution License (https://creativecommons.org/licenses/by/4.0/). 

“A caveat to this domain-specific interpretation of these results is that the missense mutations might generally perturb protein stability and/or levels which results in the distinct phenotypes we document.”

Regarding *zd144*, we state this very possibility of NMD (p. 8) and make no claims about this the phenotype being a specific effect on the C-terminal domain. In fact, we argue that it is probably a general loss of function.

The statistical analyses are appropriate for the experimental design. One-way ANOVA followed by Tukey's post hoc test was used for the analysis of VD/DD motor neuron guidance across multiple genotypes, and Fisher's exact test was applied to pairwise comparisons of AVM ventral guidance defects. The primary guidance assays included scoring of 100 animals per genotype.

The information provided allows the experiments to be reproduced. The manuscript includes descriptions of the alleles, sequence changes, and genetic backgrounds, primer sequences for amplicon sequencing and CRISPR repair templates, and publicly available RNA-seq FASTQ files. The experimental data support the conclusions regarding the differential effects of the S120F and EGF3 mutations on ventral and dorsal guidance, respectively. The genetic interaction analysis of the glycosylation-site mutants also supports the conclusion that these residues are not individually required for guidance but contribute to receptor-dependent signaling.

The methods are described in sufficient detail. The combination of EMS and ENU mutagenesis enabled the recovery of multiple allele classes. Nanopore amplicon sequencing was used to identify mutations generated in the genetic screens, and the Cas9 genome-editing procedures follow established protocols. The GFP markers juIs76 and zdIs5 were used to visualize the corresponding axons.

Several minor editorial issues should be corrected. The phrase "partially disturpted AVM ventral guidance" should be changed to "partially disrupted AVM ventral guidance."

Done.

The sentence beginning with "the zd144 deletion includes..." should begin with a capital letter.

Done.

The capitalization of "Cas9" should be used consistently throughout the manuscript.

Done.

The phrase "...detectable effect of dorsal-ventral axon guidance..." should likely read "...detectable effect on dorsal-ventral axon guidance..."

Done.

Figure labels describing the three-dimensional protein structures should be formatted consistently across all panels.

We have double-checked the figure labeling and can find no specific instance of formatting inconsistency. If we have overlooked an instance, please indicate what exactly should be changed.

Citations in the text, including Hooper et al. (2025), should be checked against the final bibliography.

We used Endnote to format the bibliography automatically. We checked the Hooper et al., 2025 citations in the text and found them to be correct. If we have overlooked an error, please indicate specifically what we should change.

© 2026. Published by The Company of Biologists under the terms of the Creative Commons Attribution License (https://creativecommons.org/licenses/by/4.0/).

Reviewer 2: This manuscript reports the isolation and characterization of fourteen new unc-6/Netrin alleles identified through genetic screens in C. elegans. The authors identify missense mutations affecting the LN, EGF1, EGF2, and EGF3 domains of UNC-6 and analyze their effects on dorsal VD/DD and ventral AVM axon guidance. In addition, CRISPR-generated mutations at three predicted N-glycosylation sites are examined alone and in sensitized genetic backgrounds.

The study represents a wide genetic structure-function analysis of UNC-6 using endogenous alleles rather than transgenic deletion constructs. The recovery of multiple independent alleles, complementation tests, rescue experiments, molecular characterization, and quantitative phenotypic analysis provide strong support for the conclusions regarding domain-specific contributions to axon guidance.

Overall, the manuscript is technically good and provides useful insights into UNC-6 domain function. However, several aspects of data presentation and interpretation could be improved.

Major comments:

1. Glycosylation-related conclusions should be moderated:

Some statements regarding N-glycosylation appear stronger than what is showed by the data. The genetic results clearly demonstrate functional consequences of mutating conserved residues that may be glycosylated. However, effects on glycosylation itself remain inferred. I recommend revising the text to distinguish between mutation of glycosylation acceptor residues, and direct evidence for glycosylation-dependent regulation. No additional experiments are necessarily required, but the interpretation should be more cautious.
Examples:
-Line 44 in the abstract: "…these glycosylation sites indeed have a role in UNC-6 signaling" consider "… that these conserved residues contribute to UNC-6 signaling."
-Line 106 in the intro "To test the role of N-glycosylation…". Glycosylation itself was not tested
-Line 110 in the intro. "glycosylation sites were eliminated", I suggest claiming "...UNC-6 function was modestly reduced when these conserved asparagine residues were mutated." Similar in final discussion paragraph starting in line 478.

Agreed. Stating that the residues are important rather than referring to them as glycosylation sites is a more conservative interpretation. We have made the changes.

Regarding the Discussion, since it is speculation, we prefer to discuss the possibility of glycosylation here, especially in the context that in the next sentence, we add a caveat about effects on protein stability as an alternate explanation.

2. Data presentation:

The valuable data present by the manuscript could benefit of a better presentation. The reading needs an uncomfortable movement cross referencing along tables and figures for each result fragment. I would suggest considering a more integrated graphical presentation like a domain-level summary figure combining (i) mutation location, (ii) phenotype severity, and (iii) affected guidance modality.

Presenting quantitative data and statistical significance is a challenge. We attempt to do what the reviewer suggests in Figure 6. We have tried other graphing formats to present the data and statistical significance, and the graphs become very complex and difficult to interpret, or very many in number. We prefer the Table to present these quantitative data.

Table 2 includes some p values express as "p < 0.0001" and others with the exact value "p = 0.0100". please clarify or homogenize this. Consider reporting: 1) exact p-values where possible rather than simply "NS", 2) measures of variance in phenotype tables and 3) graphical presentation of animal-level distributions. Although the sample sizes are appropriate, visualization of data distributions would make interpretation easier.

© 2026. Published by The Company of Biologists under the terms of the Creative Commons Attribution License (https://creativecommons.org/licenses/by/4.0/).

The *p* values come directly from the GraphPad Prism analysis, which we now include as Supplemental data. Likewise, this file contains animal-level variance of VD/DD defects. The AVM data are proportional.

Structural figures occupy substantial space relative to their role in the manuscript: Figures 2 and 5 are useful for visualizing residue locations and possible structural consequences but these panels are based on previously published structures rather than structural analyses performed in the current study. As the manuscript's primary contribution is genetic and phenotypic rather than structural. The authors may wish to consider whether some structural panels could be condensed or moved to supplementary material. Also, this figures generation are missing in the "Methods". The manuscript would benefit of clarifying how this was generated and visualized.

We prefer to keep the structures in the main manuscript. The Figure 2 legend contains details on how these images were generated and the references. We now repeat this in the Materials and Methods.

Minor comments:

3. Number of genomes screened

The manuscript would benefit from reporting the approximate scale of the EMS/ENU screens (number of genomes screened, number of F2 animals examined, and total number of axon-guidance mutants recovered). This information would provide useful context regarding screen saturation and the frequency with which unc-6 alleles were recovered.

These screens were done over many years by different people. Records of the number of genomes screened are incomplete. This information would give context but is not critical for interpretation of the results presented here.

4. Glycosylation sites. N423
The manuscript discusses N423 alongside N114 and N128, yet later notes (line 279) that N423 is unlikely to represent a bona fide glycosylation site. This distinction should be emphasized earlier to avoid confusion.

Agreed. We now state this when the residues are first discussed:
"N114 and N128 are glycosylated in the UNC-6 crystal structure, but N423 is not."

Typos and small comments to address:

- Several acronyms are not clarified the first time written: AVM, VD, DD, HSN neuron

*C. elegans* neurons were originally given somewhat arbitrary alphabetical designations. These designations are generally not spelled out in publications and rather treated as names. However, we have spelled out the acronyms to the best of our knowledge the first time they are discussed. VD/DD – ventral and dorsal D-class motor neurons; AVM = anterior ventral microtubule neuron; HSN = hermaphrodite-specific neuron.

- Line 53, "reviewed in…". Just add as one more reference.

Done.

- Line 39, in the abstract. Potential typo: "S120Y". is it "S120F"?

Done.

- line 109 extra space after mutants

Done.

© 2026. Published by The Company of Biologists under the terms of the Creative Commons Attribution License (https://creativecommons.org/licenses/by/4.0/).

- line 147 -150 different typography

Done.

- line 230: "the" ◊ The. Caps missing

Done.

- line 232 and 232 "resulting" is repeated

Done.

- line 238, reference missing on Netrin 1 in vertebrates' statement.

Done.

- line 256, again, "previously reported". Clarify where

Done.

- line 390 and line 418 use second and forth

Done.

- Clarification on line 13 on Scott G. Clark affiliation "(retired)". Check with journal how to state this.

We will work with editorial staff to ensure this is properly represented under journal standards.

---

Second decision letter

MS ID#: bio.062822R1

MS Title: Domain-specific mutations in unc-6/Netrin differentially affect dorsal-ventral axon pathfinding in Caenorhabditis elegans

Authors: Kelsey Hooper; Scott Clark; Erik Lundquist

I am happy to tell you that your manuscript has been accepted for publication in Biology Open, pending our standard publication integrity checks. Please do report any approximate information that is available regarding the scale of the mutagenesis screen in methods as requested by one of the reviewers. It was accepted on 25th August 2026.

© 2026. Published by The Company of Biologists under the terms of the Creative Commons Attribution License (https://creativecommons.org/licenses/by/4.0/).
